# LncRNA FAM83A-AS1 facilitates tumor proliferation and the migration via the HIF-1α/ glycolysis axis in lung adenocarcinoma

**DOI:** 10.7150/ijbs.67556

**Published:** 2022-01-01

**Authors:** Zhencong Chen, Zhengyang Hu, Qihai Sui, Yiwei Huang, Mengnan Zhao, Ming Li, Jiaqi Liang, Tao Lu, Cheng Zhan, Zongwu Lin, Fenghao Sun, Qun Wang, Lijie Tan

**Affiliations:** Department of Thoracic Surgery, Zhongshan Hospital, Fudan University, No. 180, Fenglin Road, Shanghai, 200032, China

**Keywords:** Lung adenocarcinoma (LUAD), Metabolic reprogramming, HIF-1α/glycolysis axis, FAM83A-AS1

## Abstract

**Background:** Lung adenocarcinoma (LUAD), the major subtype of lung cancer, is among the leading cause of cancer-related death worldwide. Energy-related metabolic reprogramming metabolism is a hallmark of cancer shared by numerous cancer types, including LUAD. Nevertheless, the functional pathways and molecular mechanism by which FAM83A-AS1 acts in metabolic reprogramming in lung adenocarcinoma have not been fully elucidated.

**Methods:** We used transwell, wound-healing scratch assay, and metabolic assays to explore the effect of FAM83A-AS1 in LUAD cell lines. Western blotting, Co-IP assays, and ubiquitination assays were used to detect the effects of FAM83A-AS1 on HIF-1α expression, degradation, and its binding to VHL. Moreover, an *in vivo* subcutaneous tumor formation assay was used to detect the effect of FAM83A-AS1 on LUAD.

**Results:** Herein, we identified FAM83A-AS1 as a metabolism-related lncRNA, which was highly correlated with glycolysis, hypoxia, and OXPHOS pathways in LUAD patients using bioinformatics analysis. In addition, we uncovered that FAM83A-AS1 could promote the migration and invasion of LUAD cells, as well as influence the stemness of LUAD cells *in vivo* and vitro. Moreover, FAM83A-AS1 was shown to promote glycolysis in LUAD cell lines *in vitro* and *in vivo*, and was found to influence the expression of genes related to glucose metabolism. Besides, we revealed that FAM83A-AS1 could affect glycolysis by regulating HIF-1α degradation. Finally, we found that FAM83A-AS1 knockdown could inhibit tumor growth and suppress the expression of HIF-1α and glycolysis-related genes *in vivo*.

**Conclusion:** Our study demonstrates that FAM83A-AS1 contributes to LUAD proliferation and stemness via the HIF-1α/glycolysis axis, making it a potential biomarker and therapeutic target in LUAD patients.

## Introduction

Lung cancer has the second-highest morbidity and the highest mortality of all malignant tumors, with a 5-year overall survival (OS) of only 18%^1^, ^2^. Lung adenocarcinoma accounts for nearly 40% of lung cancer cases, contributing to the majority of cases in terms of both incidence and mortality^3^.

Metabolic reprogramming, a hallmark of tumor cells, is an essential characteristic as it provides the energy required for survival and maintenance of cellular function^4^, ^5^. Recently, several studies found that lncRNAs, a class of non-coding transcripts that are >200 nucleotides in length, were involved in the tumor metabolic regulation process at a transcriptional level, affecting several metabolic pathways^6^, ^7^. For example, Rupaimoole R et al.^8^ identified lncRNA NRCP as an intermediate binding partner between STAT1 and RNA polymerase II, which could affect metabolism in ovarian cancer. Additionally, Huang X et al.^9^ revealed that lncRNA LINC00842 promotes PDAC malignancy through metabolic remodeling. These findings suggest that the interaction between lncRNAs and metabolic reprogramming is valuable for exploring new potential targets in cancer therapy.

LncRNA FAM83A-AS1, which has been suggested to cause and accelerate the occurrence and development of tumors^10^, ^11^, is transcribed from the antisense strand of the FAM83A gene located at 8q24.13. However, FAM83A-AS1's role in tumor development is not yet clearly defined, especially in metabolic reprogramming. This study is the first to identify and validate FAM83A-AS1 as a metabolism-related lncRNA that facilitates tumor proliferation and stemness via the HIF-1α/ glycolysis axis. Moreover, by performing Co-IP, ubiquitination, and metabolic assays we provided mechanistical insight into the role of FAM83A-AS1 in the pathogenesis of LUAD.

## Results

### Identification of FAM83A-AS1 as a metabolism-related lncRNA in LUAD patients

**Figure 1:**
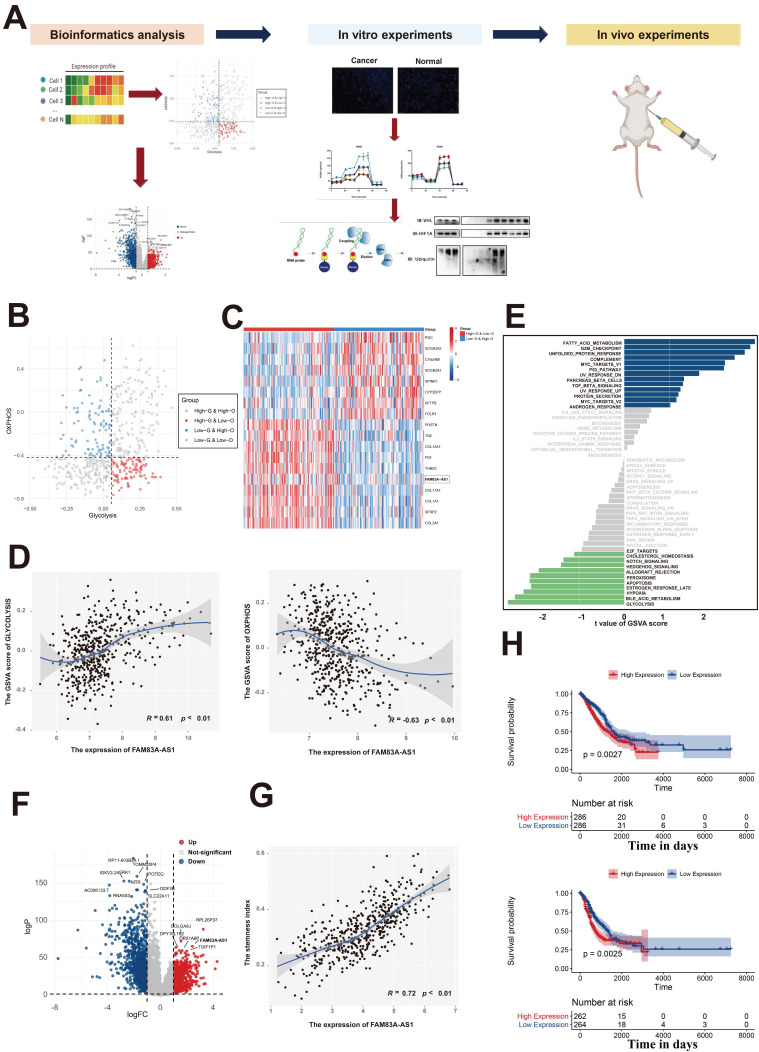
**Identification of FAM83A-AS1 as a metabolism-related lncRNA in LUAD patients.** (a) Experimental scheme for identifying FAM83A-AS1 facilitated tumor proliferation and the migration via the HIF-1α/ glycolysis axis in lung adenocarcinoma. (b) The scatter plot showing the distribution of Glycolysis score (x-axis) and OXPHOS score (y-axis) in LUAD patients. Patients were assigned to four metabolic subgroups according to the median value of the two scores. (c) Heatmap of differentially expressed genes between high-glycolysis & low-OXPHOS and low-glycolysis & high-OXPHOS groups. (d) The association between the expression of FAM83A-AS1 and energy-related metabolic pathways (glycolysis and OXPHOS pathways). (e) GSVA analysis of the hallmark pathways between low FAM83A-AS1 expression group (≤median expression level across all samples) and high FAM83A-AS1 expression group (>median expression level across all samples) in LUAD patients. (f) Differential expressed genes between the LUAD and the normal lung group were shown in a volcano plot. (g) The association between the expression of FAM83A-AS1 and stemness scores. (h) Kaplan-Meyer plot of OS (up) and DFS (down) in TCGA with high or low expression group of FAM83A-AS1.

Previous studies have reported that the rapid growth of malignant cells demands large amounts of energy, which indicates that energy-related metabolic pathways play an important role in tumorigenesis^12^-^14^. Our previous work had comprehensively analyzed the metabolic heterogeneity on a pan-cancer scale and revealed that LUAD patients could be assigned to four metabolic subgroups according to the glycolysis and oxidative phosphorylation (OXPHOS) pathways^15^ (Figure 1B). Additionally, differential expression analysis in LUAD patients between the high-glycolysis & low-OXPHOS and low-glycolysis & high-OXPHOS groups revealed that FAM83A-AS1 was significantly elevated in the high-glycolysis & low-OXPHOS groups (Figure 1C). We next sought to investigate the association between the expression of FAM83A-AS1 and energy-related metabolic pathways (glycolysis and OXPHOS pathways). As shown in Figure 1D, we found that the glycolysis activity and OXPHOS pathways were all highly correlated with the expression of FAM83A-AS1 (Pearson's R = 0.61 for glycolysis and -0.63 for OXPHOS, respectively). Moreover, in order to explore the differential activities of pathways between low FAM83A-AS1 expression group (≤median expression level across all samples) and high FAM83A-AS1 expression group (>median expression level across all samples) in LUAD patients, we performed GSVA. As shown in Figure 1E, energy-related metabolic pathways (including glycolysis, bile acid metabolism, and hypoxia) were the top 3 enriched pathways in high FAM83A-AS1 expression group, suggesting FAM83A-AS1 could serve as a metabolism-related lncRNA in LUAD patients.

Next, to explore the oncogenic role of FAM83A-AS1 in LUAD patients, we performed differential expression analysis in LUAD tissues and normal lung tissues of TCGA data using the edgeR package. As shown in Figure 1F, we observed that FAM83A-AS1 was significantly elevated in LUAD tissues. Moreover, our previous study^16^ revealed that the stemness index could serve as a biomarker in LUAD. Therefore, the relationship between the expression of FAM83A-AS1 and stemness scores was also investigated in our study. Intriguingly, as shown in Figure 1G, we found that stemness scores were positively correlated with FAM83A-AS1 expression in TCGA-LUAD patients. Additionally, we also evaluated the association between the FAM83A-AS1 expression and the clinicopathological features in LUAD patients and our results revealed that the higher expression level of FAM83A-AS1 was also linked with a worse prognosis (Figure 1H). Additionally, our results also suggested that FAM83A-AS1 expression was strongly associated with TNM staging, tumor graded, tumor size, and distant metastasis (Supplementary Figure 1A and Supplementary Figure 1B). On the other hand, differential analysis in the somatic mutation distribution was also performed in our study. As shown in Supplementary Figure 1C, our study revealed distinct patterns of the mutation between the high FAM83A-AS1 expression group and low FAM83A-AS1 expression group. Overall, our results reveal that FAM83A-AS1 is a metabolism-related lncRNA in LUAD patients and is vital for the LUAD.

### LncRNA FAM83A-AS1 promotes the migration, invasion and stemness of LUAD cell lines under hypoxic conditions *in vitro*

**Figure 2:**
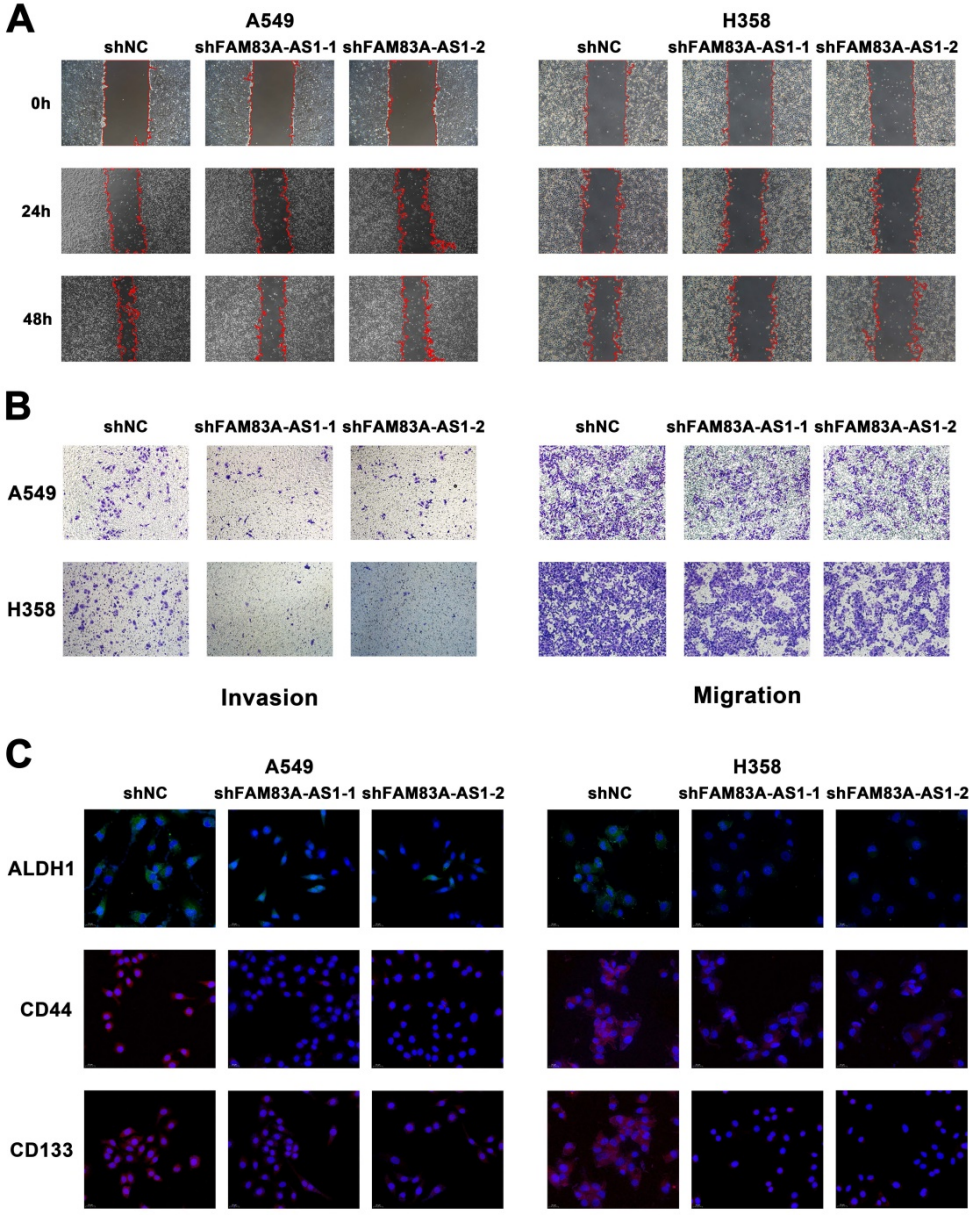
** LncRNA FAM83A-AS1 promotes the migration, invasion and stemness of LUAD cell lines under hypoxic conditions *in vitro*.** (a) Wound-healing scratch assays of FAM83A-AS1 knockdown and control LUAD cells under hypoxic conditions. (b) Cell migration and invasion assays were performed in FAM83A-AS1 knockdown and control LUAD cells under hypoxic conditions. (c) Fluorescence *in situ* hybridization of stemness-related genes in FAM83A-AS1 knockdown and control LUAD cells under hypoxic conditions.

To investigate the possible role of lncRNA FAM83A-AS1 in lung adenocarcinoma, we used wound-healing scratch assays to determine its effect on the invasion and migration ability of A549 and H358 cell lines. We found that FAM83A-AS1 knockdown could barely reduce the migration ability of LUAD cells under a normoxia environment (Supplementary Figure 2A). However, considering the close relationship between FAM83A-AS1 and hypoxia, we repeated the above experiments under hypoxic conditions. Interestingly, results showed that knockdown of FAM83A-AS1 in both A549 and H358 cell lines significantly inhibited the invasion and migration abilities of LUAD treated with 1% O_2_ (Figure 2A). In addition, we also performed transwell assays in normal and FAM83A-AS1 knockdown cell lines under hypoxic conditions. As expected, the results were in line with our above outcomes (Figure 2B). On the other hand, we also found that compared with lung adenocarcinoma cells that did not knock out FAM83A-AS1, the proliferation of knocked out cells was significantly inhibited (Supplementary Figure 2B).

We further explored the relationship between FAM83A-AS1 and cell stemness. Using quantitative real-time PCR, we measured the expression of cell stemness-related genes in A549 and H358 cell lines depleted of or overexpressed with FAM83A-AS1. The results revealed that CD133, CD44, and ALDH1 expression levels were decreased in both cell lines when FAM83A-AS1 was depleted, while they were elevated in the FAM83A-AS1 overexpressed cell lines (Supplementary Figure 2C). In addition, CD33 showed a similar trend in H358 cells but not in A549 cells. Then, to further verify the effect of FAM83A-AS1 on stemness-related genes, we explored the amplification variations of the above genes using fluorescence *in situ* hybridization (FISH) experiments. Similarly, the results confirmed that amplification of the above genes was enhanced in FAM83A-AS1 overexpressed cells, but little amplification was seen in FAM83A-AS1 knockdown cells (Figure 2C). In addition, flow cytometry also confirmed reduced CD133 expression in FAM83A-AS1 knockdown A549 cells (Supplementary Figure 2D).

Collectively, the above experimental results confirmed that inhibition of lncRNA FAM83A-AS1 expression reduced the invasion and migration abilities of lung adenocarcinoma and FAM83A-AS1 could impact the cell stemness by influencing the expression of CD133, CD44, and ALDH1 in LUAD under hypoxic conditions.

### LncRNA FAM83A-AS1 promotes glycolysis in LUAD cell lines *in vitro* and *in vivo* and influences the expression of genes related to glucose metabolism

**Figure 3:**
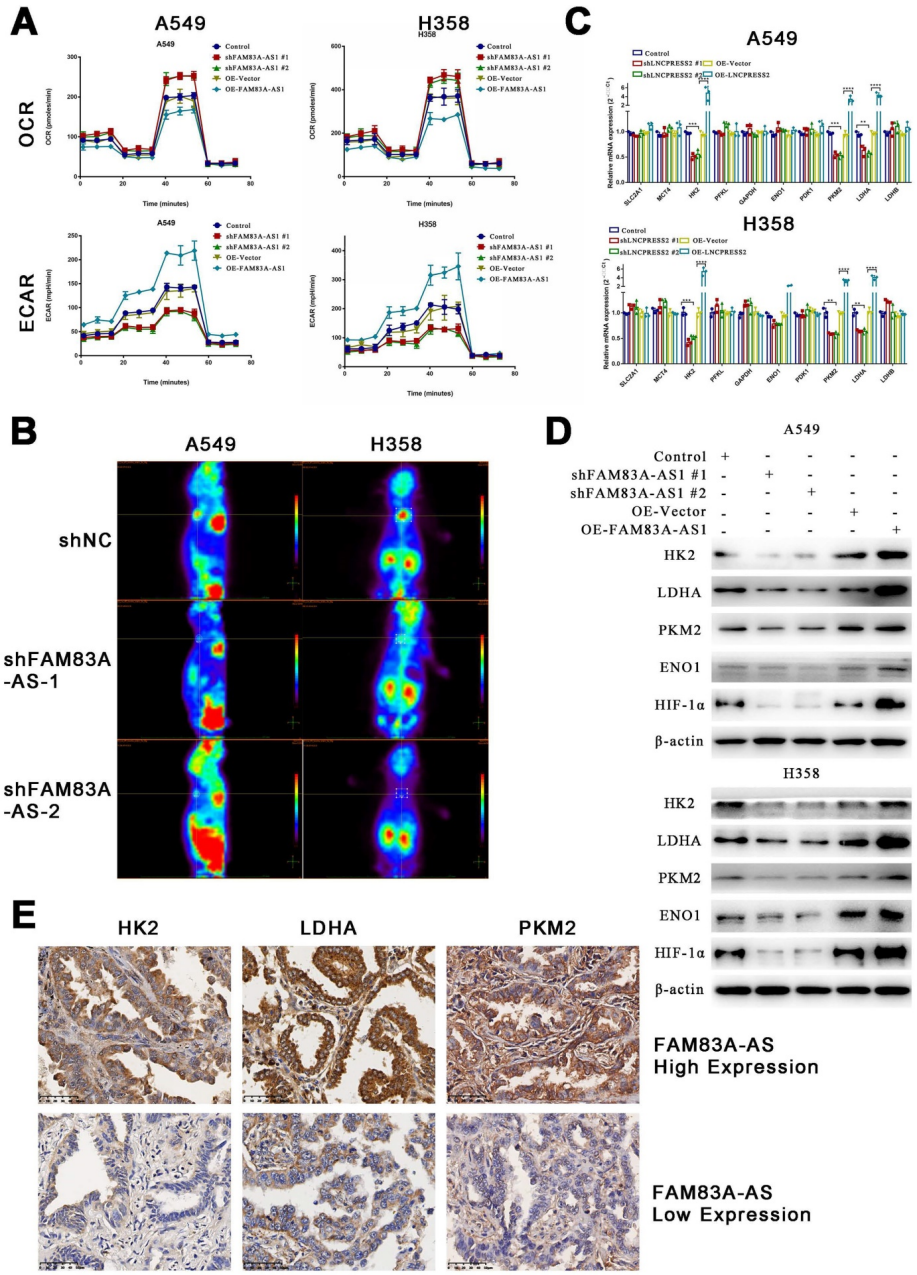
** LncRNA FAM83A-AS1 promotes glycolysis in LUAD cell lines *in vitro* and *in vivo* and influences the expression of genes related to glucose metabolism.** (a) The O2 consumption rate (OCR) and extracellular acidification rate (ECAR) of FAM83A-AS1 knockdown, overexpressing and control cells. (b) PET/CT of mouse model which subcutaneous injected FAM83A-AS1 knockdown and control A549 cells. (c) Relative gene expression(2-ΔΔCT) of glucose metabolism-related genes in FAM83A-AS1 knockdown, overexpressing and control cells. (d) Western blotting detected the expression levels of glucose metabolism-related genes and HIF1A in FAM83A-AS1 knockdown, overexpressing and control cells. (e) Immunohistochemistry of glycolysis-related genes expressed in patients of different FAM83A-AS1 expression LUAD.

FAM83A-AS1 has been proven to be involved in cellular metabolic processes such as glycolysis and oxidative phosphorylation previously. To further explore the relationship between FAM83A-AS1 and metabolism, we used the Seahorse assay to determine the metabolic alterations in knockdown or overexpressed FAM83A-AS1 LUAD cell lines. The O_2_ consumption rate (OCR) showed that the basal respiration of the cells was slightly reduced, while maximal respiratory capacity was significantly inhibited in cells overexpressing FAM83A-AS1 (Figure 3A). The extracellular acidification rate (ECAR) significantly increased glycolysis and glycolytic capacity in cells overexpressing FAM83A-AS1. As expected, the opposite trend was observed in FAM83A-AS1 knockdown cells. The above results suggest that FAM83A-AS1 can inhibit oxidative phosphorylation and promote cellular glycolytic capacity.

Furthermore, to further verify the effects of FAM83A-AS1 on the metabolism *in vivo*, we subcutaneously injected FAM83A-AS1 overexpressed or knocked down A549 cells into nude mice. After two weeks, PET/CT of the xenografts showed that tumors formed in the FAM83A-AS1 overexpressed group were significantly larger and their metabolic capacities were increased compared to the FAM83A-AS1 knockdown group (Figure 3B). In summary, our study confirms that FAM83A-AS1 can affect tumor metabolism, especially enhancing LUAD cells' glycolysis *in vitro* and *in vivo*.

Next, we verified the expression of glucose metabolism-related genes using qRT-PCR in cell lines overexpressing or depleted of FAM83A-AS1. We found that HK2, PKM2, and LDHA were highly expressed in FAM83A-AS1 overexpressing cells, while a down-regulation was observed in FAM83A-AS1 knocked down cells (Figure 3C). Then, we verified the expression of the above three genes and hypoxia-inducible factor 1 subunit alpha () at the protein level. The results demonstrated that FAM83A-AS1 overexpression could promote the protein expression of the above genes, while the opposite results were observed after FAM83A-AS1 knockdown (Figure 3D). Consistently, we observed similar results in patient's samples with high expression of FAM83A-AS1 (Figure 3E). Thus, we hypothesized that FAM83A-AS1 could affect LUAD metabolism by regulating the expression of genes related to glucose metabolism.

### LncRNA FAM83A-AS1 binds to HIF-1α at its N-terminal VHL recognition site, thereby inhibiting HIF-1α degradation by obstructing the interaction of HIF-1α with VHL

To further investigate our above results, we used PRIDB (http://bindr.gdcb.iastate.edu/PRIDB)^17^ to predict the proteins that can bind to FAM83A-AS1. We found that FAM83A-AS1 was able to bind to HIF-1α with a binding probability of 0.80. Next, we used catRAPID (http://service.tartaglialab.com/page/catrapid_group)^18^ to predict the potential binding sites between HIF-1α and FAM83A-AS1. The results showed that the binding region was probably located at the 376-527 amino acid sites of HIF-1α (Supplementary Table 1). We found that this locus overlaps with the pVHL binding site. Under normoxia, the oxygen-dependent degradation (ODD) region of HIF-1α is recognized and hydroxylated by PHD enzymes^19^, ^20^. After hydroxylation, HIF-1α can bind to pVHL and undergoes ubiquitination and subsequent degradation by the proteasome^21^. Therefore, we hypothesized that FAM83A-AS1 could competitively bind at the binding site of pVHL on HIF-1α, preventing the attachment of pVHL and eventually inhibiting the degradation of HIF-1α.

**Figure 4:**
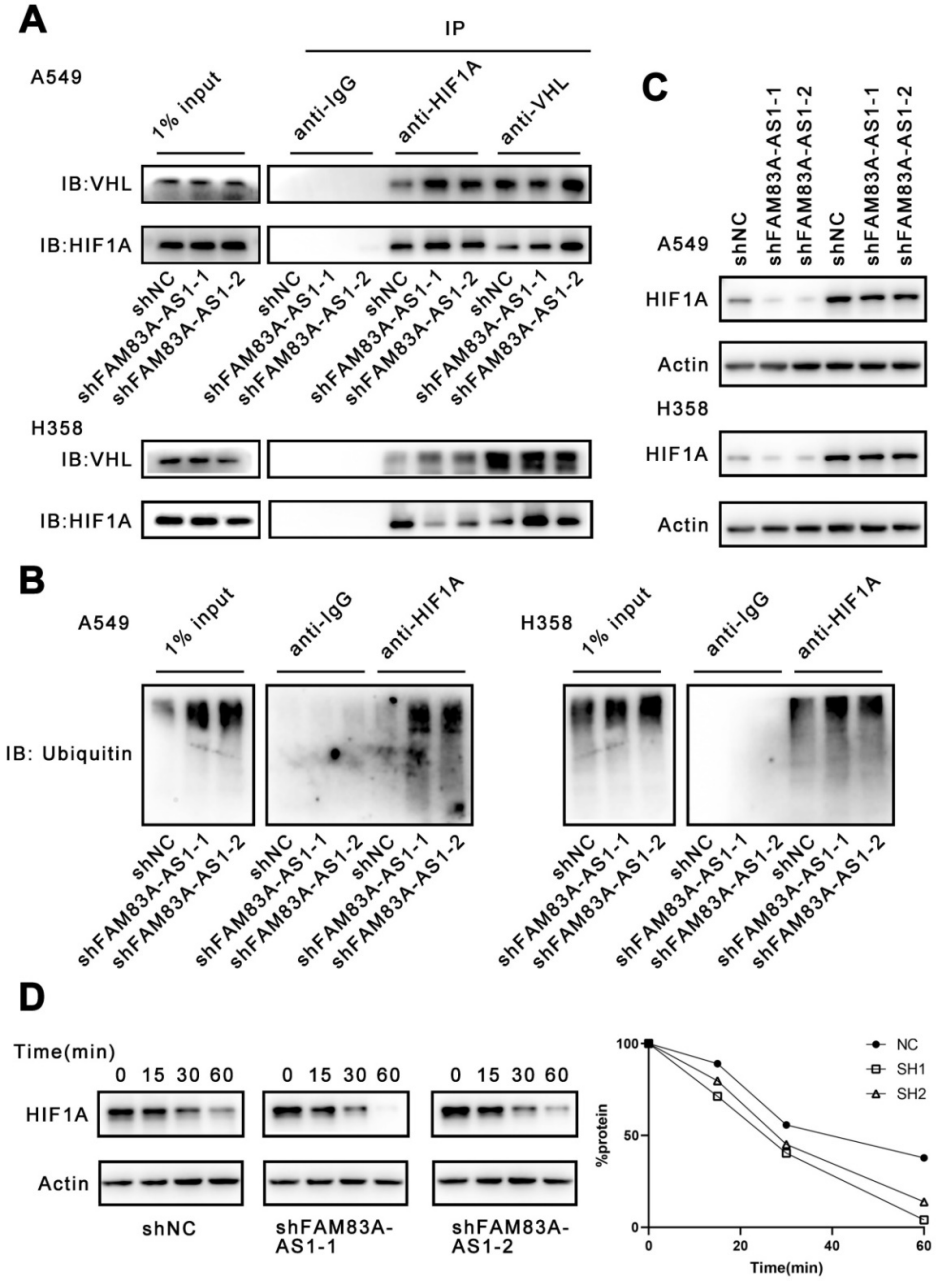
** LncRNA FAM83A-AS1 inhibits HIF-1α degradation by obstructing the interaction of HIF-1α with VHL.** (a) Immunoprecipitation of anti-HIF1A and anti-VHL in hypoxia treated A549 and H358 cells knocking down FAM83A-AS1. (b) The ubiquitination level of HIF1A in hypoxia conditions of FAM83A-AS1 knockdown and control A549 and H358 cells. (c) The HIF1A expression level was analyzed by western blotting in FAM83A-AS1 knockdown and control LUAD cells after treated with proteasome inhibitor MG132 (10μM). (d) The HIF1A expression level was analyzed by western blotting in FAM83A-AS1 knockdown and control LUAD cells after treated with 25 μM CHX for indicated time periods under normoxic conditions.

Considering that FAM83A-AS1 could attach to the binding site of HIF-1α and VHL, we investigated whether FAM83A-AS1 could affect the interaction of HIF-1α and VHL. Our results demonstrated that FAM83A-AS1 knockdown could promote the interaction of HIF-1α and VHL in LUAD cell lines (Figure 4A). HIF-1α can form a complex with Cullin-RING E3 ubiquitin ligase (CRLs) upon combining with VHL and is subsequently degraded by the proteasome^22^-^24^. However, under normoxic conditions, HIF-1α is not recognized by VHL and thus accumulates intracellularly. Therefore, we next detected the ubiquitination level of HIF-1α. The results revealed that FAM83A-AS1 knockdown caused a significant increase in the ubiquitination level of HIF-1α (Figure 4B). Subsequently, we treated the A549 and H358 cells with the proteasome inhibitor MG132 and assayed the expression level of HIF-1α. We found that MG132 almost eliminated the inhibitory effect of FAM83A-AS1 on HIF-1α (Figure 4C). To investigate whether FAM83A-AS1 affects the stability of HIF-1α, we treated the cells with cycloheximide (CHX), an inhibitor of protein synthesis. Cells were first incubated under hypoxic conditions for 24 hours, followed by the addition of CHX and resumption of normoxic conditions. Compared with normal LUAD cells, the half-life of HIF-1α was significantly shorter in FAM83A-AS1 knockdown cells (t1/2 shNC vs. sh FAM83A-AS1 vs. shFAM83A-AS2 = 37 vs. 24 v.s 22 min, *p* < 0.05) (Figure 4D). In summary, we reveal that FAM83A-AS1 can inhibit the binding of HIF-1α to VHL, weakening VHL-mediated ubiquitination and proteasomal degradation, which in turn leads to the accumulation of HIF-1α.

### The effect of LncRNA FAM83A-AS1 on migration and invasion of LUAD cells is regulated by HIF-1α

**Figure 5:**
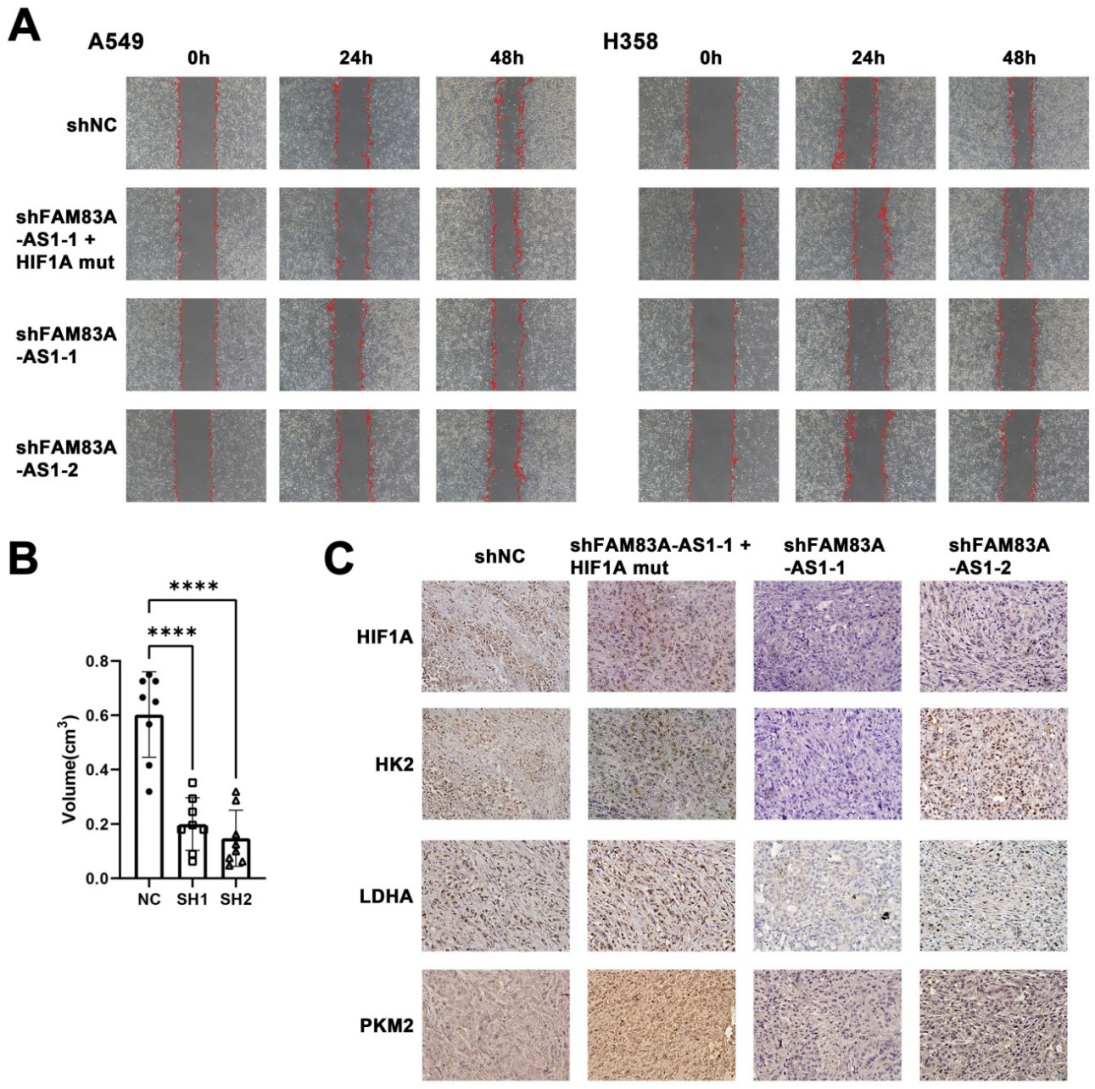
** The effect of LncRNA FAM83A-AS1 on migration and invasion of LUAD cells is regulated by HIF1A and knockdown LncRNA FAM83A-AS1 inhibits tumor growth and suppresses the expression of HIF-1α and glycolysis-related genes *in vivo*.** (a) Wound-healing scratch assays of FAM83A-AS1 knockdown and control LUAD cells and FAM83A-AS1 knockdown cells transfected with the mutated HIF-1α plasmid under hypoxic conditions. (b) Tumor volume size of tumors in subcutaneous mouse model which injected FAM83A-AS1 knockdown and control LUAD cells. (c) Immunohistochemistry of glycolysis-related genes and HIF1A expressed in tumors from subcutaneous mouse model.

Next, we confirmed whether FAM83A-AS1 exerts its biological role through HIF-1α. To that end, we transfected HIF-1α plasmids, whose VHL binding sites have been mutated in FAM83A-AS1 knockdown cells. We found that FAM83A-AS1 knockdown cells transfected with the mutated HIF-1α plasmid had a significantly subdued migration and invasion compared to FAM83A-AS1 knockdown cells alone, with a trend resembling that of normal cells (Figure 5A). This suggests that FAM83A-AS1 promotes the invasion and migration of LUAD cells by mediating the high expression of HIF-1α.

### Knockdown of LncRNA FAM83A-AS1 inhibits tumor growth and suppresses the expression of HIF-1α and glycolysis-related genes *in vivo*

To investigate if the effects of FAM83A-AS1 would translate *in vivo*, we used nude mice injected with A549 cells transfected with shFAM83A-AS1 or a control vector. Mice were sacrificed 4 weeks later, and tumors were dissected and measured. The results revealed that the tumor volume of the control group transfected with the blank vector was significantly larger than that of the knockdown FAM83A-AS1 group (Figure 5B, Supplementary Figure 2). Next, the tumor tissues were sectioned and stained, and similarly, the results confirmed high expression of HIF-1α and glycolysis-related genes like LDHA, HK2, PKM2 in the control group and low expression in the shFAM83A-AS1 LUAD (Figure 5C). In conclusion, our results confirmed that FAM83A-AS1 could promote LUAD development *in vivo* by affecting HIF-1α and enhancing glycolysis.

## Discussion

**Figure 6:**
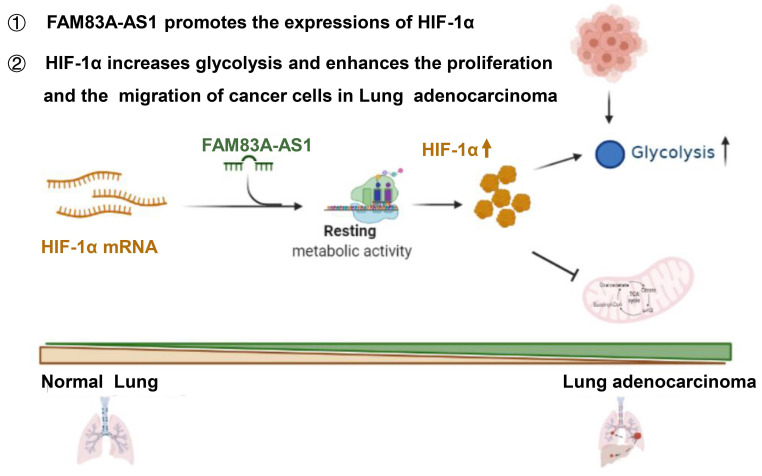
Schematic model of FAM83A-AS1-HIF-1α signaling axis in LUAD.

Herein, we are first to identify FAM83A-AS1 as a metabolism-related lncRNA, which was found to be highly correlated with glycolysis, hypoxia, and OXPHOS pathways in LUAD patients using bioinformatics analysis. Subsequent functional experiments revealed that FAM83A-AS1 promotes invasion, migration and enhances cell stemness in LUAD cells under hypoxic conditions. In addition, FAM83A-AS1 could promote glycolysis and affect the expression of glycolysis-related genes *in vitro* and *in vivo*. Our study further uncovered that FAM83A-AS1 could bind to the N-terminal of HIF-1α to inhibit the interaction between HIF-1α and VHL, thus preventing the degradation of HIF-1α and allowing its stable presence intracellularly. In contrast, transfecting with the mutated HIF-1α plasmid in cells with knockdown of FAM83A-AS1 eliminated the migration and invasion changes. In summary, our study demonstrates that FAM83A-AS1 contributes to LUAD development via the FAM83A-AS1/HIF-1α/glycolysis axis (Figure 6).

Many studies have reported that lncRNA, FAM83A-AS1 has potent tumor-promoting activity in oesophageal squamous cell carcinoma (ESCC), lung cancer, and liver carcinoma^25^-^27^. For instance, Xiao G et al. 28 discovered that FAM83A-AS1 could promote LUAD cell migration and invasion, consistent with our results. In addition, several research showed that lncRNAs play a major role in inhibiting drug resistance and act as mediators involved in different chemoresistance mechanisms^29^. Previous studies confirmed that FAM83A-AS1 could promote the expression of intracellular FAM83A and thus confers tumor cells with EGFR-TKI resistance^30^-^32^, indicating that targeting FAM83A-AS1 might have the potential to reverse EGFR-TKI resistance in LUAD. Overall, these results suggest that FAM83A-AS1 is a potential modulator of cancer driver genes and a prognostic marker across different tumors.

The role of lncRNAs in tumor development and metabolism have received increasing attention^33^.For example, the lncRNA MALAT1, UCA1, NBR2, etc. has been reported to regulate tumor cell metabolism, including promotes a glycolytic phenotype and increased lactate production, through regulating metabolic transcription factor, enzymes and relating pathways^34^-^36^. HIF-1α-stabilizing lncRNA (HISLA) from tumor-associated macrophages regulates aerobic glycolysis in breast cancer cells by inhibiting the hydroxylation and degradation of HIF-1α. Thus, the understanding of the role of lncRNAs in metabolism may help provide new therapeutic targets and novel diagnostic and prognosis markers for human cancer.

LncRNAs that take effect via reducing the intracellular transcripts level, or attenuating their activities and molecular functions in malignant cells^37^ are potential targets for cancer therapy. For example, MALAT are associated with tumor recurrence after liver transplantation^38^, as well as suppresses the progression and metastatic ability of breast cancer cells^39^ . H19 is re-expressed during tumorigenesis and promoted tumorigenic properties in broad tissue types including breast, lung, esophageal and bladder^37^. Moreover, a clinical trial from Wuhan Union Hospital on lncRNA as a potential target for lung cancer diagnosis is underway. This clinical trial is mainly based on the identification of early lung-cancer-specific exosomal lncRNA biomarkers to improve the diagnosis rate of early lung cancer^40^. Also, a clinical trial which is related to HOTAIR and thyroid cancer is being processed^41^. Furthermore, numerous studies recent years have demonstrated that lncRNAs play a significant role in resistance of cisplatin and oxaliplatin based chemotherapy 42, 43. These all imply that targeting lncRNAs may show important clinical implication by selectively affecting disseminated cancer cells or residual cancer cells after surgery. Unfortunately, till now, none of the drugs directly targeting lncRNA were approved for application of anti-tumor therapy.

Moreover, our present study found evidence of the critical role of a specific lncRNA in the positive feedback loop of assembling the HIF-1α transactivation complex, which is similar to previous studies^44^. For instance, lincRNA-p21 was reported as a hypoxia-sensitive lncRNA by influencing the stability of HIF-1α protein. This positive feedback loop reciprocally promoted glycolysis under hypoxia^45^. Another study showed that hypoxia-inducible lncRNA (LncHIFCAR)^46^ formed a complex with HIF-1α via direct binding and facilitated the recruitment of HIF-1α to the target promoters, thus was crucial for metabolic shifting and progression of oral carcinoma^47^.

On the other hand, although multiple treatment strategies have been used in LUAD patients, the overall prognosis of LUAD remains poor^48^, ^49^, indicating new therapeutic approaches that are more effective in treating LUAD are still warranted. Recently, many studies have demonstrated that understanding metabolic reprogramming of tumor cells is fundamental for understanding tumor drug resistance and developing anticancer therapy^50^, ^51^. Malignant cells generally obtain energy through glycolysis, which is less efficient rather than mitochondrial oxidative energy metabolism, which produces more energy but produces the byproduct, lactic acid^52^, ^53^. Previous studies demonstrated that the acidic TME could attenuate anti-tumor lymphocytes' ability to suppress tumor activation^54^, ^55^. The rapid progress of malignant cells combined with abnormal angiogenesis results in hypoxia within the tumor microenvironment, which leads to the accumulation of low oxygen-responsive HIF-1α 56, 57. Furthermore, upregulation of HIF-1α activates many vital metabolism-related cancer marks such as angiogenesis, glucose metabolism and cell proliferation/viability, which has a crucial role in tumor survival and progression, leading to the increased possibility of metastasis as well as mortality rates clinically^58^. Previous studies proved that selective HIF-1α targeting molecules are highly likely to be the focus of future research^58^, including mTOR inhibitors, cardiac glycosides and topoisomerase 59.

Nevertheless, our study has some limitations. Firstly, we did not clarify the specific effects of FAM83A-AS1 on cell proliferation and stemness. Moreover, the regulation mechanism of FAM83A-AS1 on glycolysis-related genes was not particularly defined, which we aim to clarify in our further research. Overall, our study demonstrates that FAM83A-AS1 is a potential biomarker and therapeutic target in LUAD. Importantly, we uncovered that FAM83A-AS1 contributes to LUAD development via the FAM83A-AS1/HIF-1α/glycolysis axis. Our findings offer new insight into the association between lncRNAs and metabolism remodeling, providing novel targets for cancer therapy.

## Materials and methods

### Ethics statement

Approval for this study was issued by the Ethics Committee of Zhongshan Hospital, Fudan University, China (B2021-137R). Patients gave informed consent upon hospitalization.

### Patients and specimens

Tissue samples were obtained from patients who underwent surgery in the Department of Thoracic Surgery, Zhongshan Hospital, between January 2019 and December 2020 and whose postoperative pathology was confirmed as lung adenocarcinoma. All studies on human specimens were approved by the Ethics Committee of Fudan University Zhongshan Hospital.

### Identification of FAM83A-AS1 as a metabolism-related lncRNA with Bioinformatic analysis

The expression and clinical data of FAM83A-AS1 in TCGA-LUAD patients were downloaded from the TCGA data portal (https://xenabrowser.net/). Maftools package^60^ was used to summarized, analyzed, annotated, and visualized the somatic mutations and copy number variants in our research. GSVA package^61^ was applied in our study to calculate the metabolic pathway scores. The gene sets of pathways were downloaded from The Molecular Signatures Database (MSigDB) (http://software.broadinstitute.org/gsea/msigdb/index.jsp). Pathway-level-metabolic gene set enrichment analysis was performed using R Bioconductor package GSVA v1.32.0 function gsva() with parameters “method = gsva, rnaseq = FALSE, abs.ranking = FALSE, min.sz.

### Cell culture and lentivirus infection

The lung adenocarcinoma cell lines (A549, H358) were purchased from the Chinese Academy of Sciences Cell Bank. Cells were cultured in DMEM (Hyclone, UT, USA) supplemented with 10% fetal bovine serum (Hyclone, UT, USA), 1% penicillin, and 1% streptomycin cultured at 37°C in a 5% CO2 atmosphere and saturated humidity. The hypoxic environment was simulated by 94% N_2_, 1%O_2_, and 5% CO_2_. Two different short hairpin RNAs (shRNA) targeting FAM83A-AS1 were ligated to lentiviral vectors with puromycin resistance, transfected using lipo3000 (Invitrogen, CA, USA), and cell lines were subsequently screened with puromycin.

### Quantitative real-time polymerase chain reaction (qRT-PCR)

RNA was extracted with TRIzol (Invitrogen, CA, USA), and PrimeScript RT Reagent kit (TaKaRa, Tokyo, Japan) was used for reverse transcription. SYBR Green Premix Ex Taq (TaKaRa, Tokyo, Japan) was used to perform qRT-PCR analysis. Relative RNA expression levels were all measured by the QuantStudio 5 Real-Time PCR System (Thermo Fisher Scientific, MA, USA). The primer sequences were provided in supplementary materials.

### Western blotting

Western-blot assay was used to analyze protein expression. The following antibodies were used to assay: anti-HIF-1α (1:1000, #36169, Cell Signaling Technology), anti-PKM2 (1:1000, #4053, Cell Signaling Technology), anti-LDHA (1:1000, #3582, Cell Signaling Technology), anti-Hexokinase II (1:1000, #ab209847, Abcam), anti-Enolase-1 (1:1000, #3810, Cell Signaling Technology), anti-VHL (1:1000, #68547, Cell Signaling Technology), anti-Ubiquitin (1:1000, #3936, Cell Signaling Technology), anti-β-ACTIN (1:3000, #AF5001, Beyotime). The band intensity was quantified using Image J software (NIH, Bethesda).

### Cell migration and invasion assays

A Transwell system (8 μm pore size, BD Biosciences) was used for cell migration and invasion assays. Cells were seeded in the upper chamber and cultured in a serum-free medium, and the lower chamber was supplemented with serum medium. For cell invasion assay, the bottom of the upper chamber was coated with Matrigel. After 48 hours of cell seeding, cells on the upper surface were swabbed off, followed by fixing, staining, washing of the cells on the lower surface, and photographed for counting. The wound-healing scratch assay was also used to test the ability of cellular migration. Cells were seeded in a six-well plate one day in advance. Cells were scratched using a 20 μl pipette tip, and the cells were incubated in a serum-free medium for 72 hours. Scratch wounds were monitored and photographed at 24, 48, and 72 hours. The distance between the two edges of the scratch wound was measured using Image J software.

### CCK8 assay

The required cells were plated in a 96-well plate with 2000 cells per well, the day before the experiment. After the cells adhered, CCK-8 was added to the wells, and the absorbance was measured after incubating for 2 hours at 37℃. The absorbance was measured after 24, 48, and 72 hours.

### Co-IP assays

Cells were lysed, and proteins were extracted using Cell lysis buffer for Western and IP (#P0013, Beyotime). We utilized anti-HIF-1α (1:50, #36169, Cell Signaling Technology) and anti-VHL (1:50, #68547, Cell Signaling Technology) antibodies for pull-down assays at 4°C and 20rpm overnight. Protein A+G Agarose (#P2055, Beyotime) was then added and mixed at 4°C, 20 rpm for 4 hours. The precipitate was centrifuged, washed and mixed with SDS-PAGE sample loading buffer (#P0015A, Beyotime) and denatured at 100°C for 10 min. The rabbit purified IgG was used as a negative control.

### Ubiquitination assays

Cells were treated with the proteasome inhibitor MG132 (10 μM) for 8 h. HIF-1α, and its binding protein were pulled down using the Co-IP assay as described above, and ubiquitination levels were detected using anti-Ubiquitin (1:1000, #3936, Cell Signaling Technology) antibodies.

### Metabolic assays

The XFe96 metabolic analyzer (Seahorse, Agilent Technologies, Santa Clara, USA) was used to detect the metabolic state of the cells. Distilled water was added to the sensor cartridge the day before the experiment and incubated overnight at 37 °C in a non-CO2 incubator. A549 and H358 cells were plated on Seahorse XF96 plates (Agilent Technologies) with a density of 5 x 104 cells per well overnight. On the day of the experiment, the distilled water was replaced with XF calibration solution in the sensor cartridge and the media in the Seahorse XF96 plates was changed to Assay Media. Sensor cartridge and Seahorse XF96 plates were both incubated for one hour at 37°C in a non-CO2 incubator. Glycolytic capacity was measured after injection of Glucose, Oligomycin and 2-DG. Glucose oxygen consumption rate was measured after injection of Oligomycin, FCCP and Rotentone/Antimycin A. Oxygen consumption rates (OCR) and extracellular acidification rates (ECAR) were also measured. Background OCR and ECAR were derived from wells without cells (medium only) and were automatically subtracted by the software.

### Immunohistochemistry

Immunohistochemistry was performed according to standard protocols^62^. Rabbit anti- HIF-1α (1:100, #ab51608, abcam), rabbit anti- Hexokinase II (1:500, #ab209847, abcam), rabbit anti- LDHA (1:500, #3582, Cell Signaling Technology), rabbit anti- PKM2 (1:800, #4053, Cell Signaling Technology) antibodies were used.

### Fluorescence *in situ* hybridization

The Cy3-labeled CD133, CD44, ALDH1 probe was constructed by RiboBio (Guangzhou, China). Fluorescence signals were generated using a Fluorescence *In situ* Hybridization Kit (RiboBio, China), and a Nikon A1 confocal laser scanning microscope (Nikon, Japan) was utilized to take pictures. Fluorescence was quantified using ImageJ (ver. 1.32j, NIH).

### Flow Cytometry

Cells and FITC-conjugated mouse anti-human CD133 (5 μL/106 cells; cat. no.: 567033, BD Biosciences) were incubated on ice for 30 minutes. Then, FACSAria III (BD Biosciences) was used to quantify the required cells, and FlowJo software (TreeStar, Woodburn, OR, USA) was used to analyze the results.

### Subcutaneous tumor formation assay

Male BALB/c nude mice (6-weeks-old) (Shanghai Slac Laboratory Animal Co., Ltd., Shanghai, China) were maintained in laminar flow cabinets. Nude mice were subcutaneously inoculated with 200μL (1 × 106) A549 cells transfected with a blank vector or shFAM83A-AS1 vector. The mice were sacrificed 4 weeks later under anesthesia, and the tumors were measured and stained in polymethylene for further analysis.

### Statistical analysis

R software 3.63, SPSS software 25.0, and Graphpad Software 9.0 were used to assess data. Data are presented as the mean ± standard error of the mean (SEM). Student's t-test was used to compare the differences between two groups. Correlations between two variations were analyzed by Pearson's correlation. Kaplan-Meier survival curves and log-rank test were employed to depict overall survival (OS). The differences of tumor volume among groups were analyzed by analysis of variance (ANOVA), followed by two-tailed Student's t test. The statistical significances of groups are represented as *p < 0.05, ** p < 0.01, *** p < 0.001, and **** p < 0.0001.

## Supplementary Material

Supplementary figures and information.

Supplementary table.
