# Supplementary material for: LncRNA FAM83A-AS1 facilitates tumor proliferation and the migration via the HIF-1α/ glycolysis axis in lung adenocarcinoma
Source: Int J Biol Sci. 2022 Jan 1;18(2):522–35. doi: 10.7150/ijbs.67556 (PMC8741836; doi:10.7150/ijbs.67556)
Supplement: Supplementary file 1 — Supplementary figures and information. [file ijbsv18p0522s1.pdf]

Supplementary Figure 1

(a). The association between the expression of FAM83A-AS1 and tumor size.

(b). The association between the expression of FAM83A-AS1 and AJCC stage.

(c) . The association between the expression of FAM83A-AS1and somatic mutation distribution

Supplementary Figure 2

(a)Wound-healing scratch assays of FAM83A-AS1 knockdown and control LUAD cells under normoxic conditions.

(b) Growth curves of A549 and H358 cell lines were measured after transfection with lentivirus vectors by CCK-8.

(c) Relative gene expression($2^{-\Delta\Delta CT}$) of stemness-related genes in FAM83A-AS1 knockdown, overexpressing and control cells.

(d) The expression of CD133 in FAM83A-AS1 knockdown and control LUAD cells using Flow cytometric.

Supplementary Figure 3. Subcutaneous tumorigenesis assay shows tumor size in mice injected FAM83A-AS1 knockdown and control A549 cells.

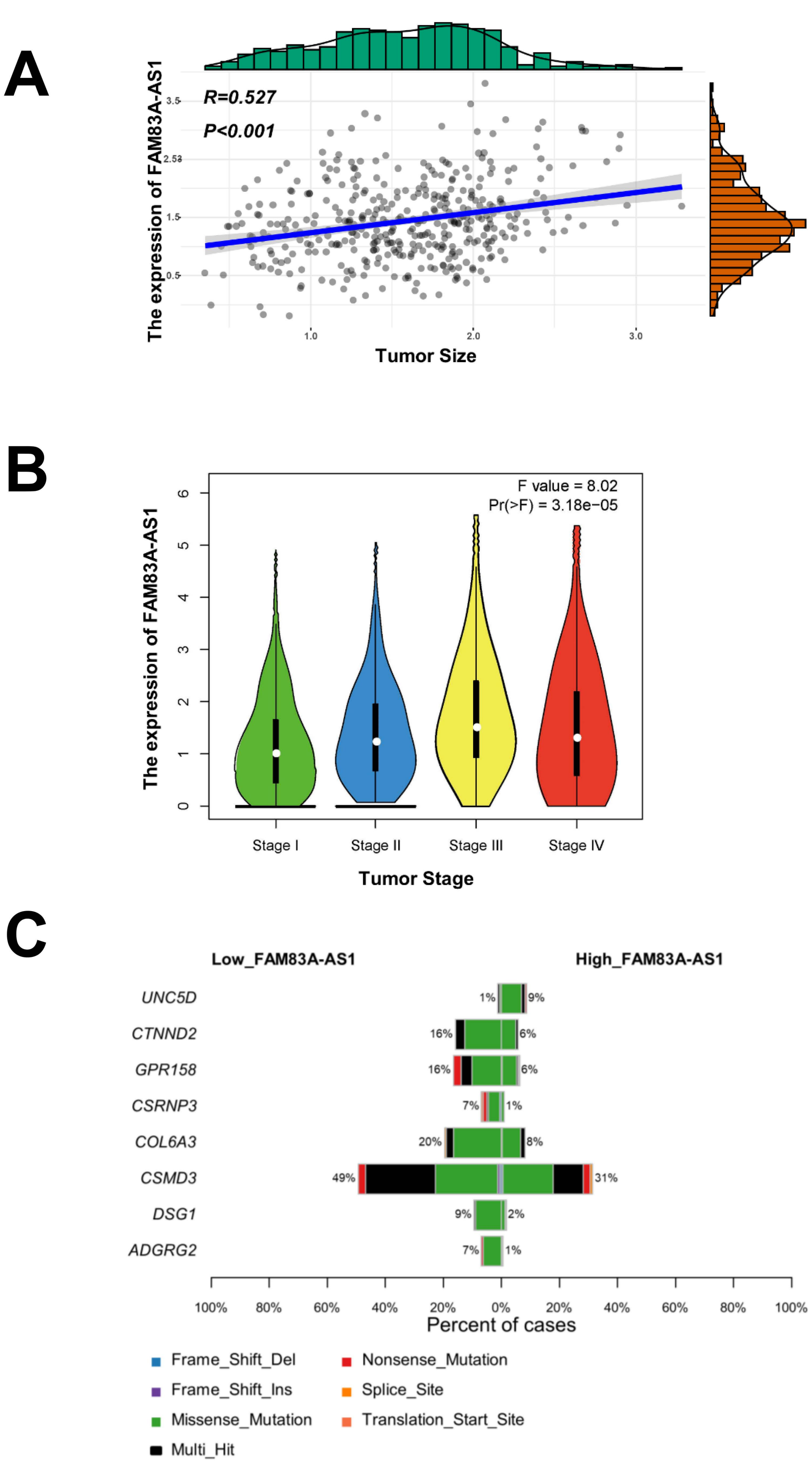
A
R=0.527
P<0.001
The expression of FAM83A-AS1
Tumor Size
B
F value = 8.02
Pr(>F) = 3.18e−05
The expression of FAM83A-AS1
Stage I
Stage II
Stage III
Stage IV
Tumor Stage
C
Low_FAM83A-AS1
High_FAM83A-AS1
UNC5D
CTNND2
GPR158
CSRNP3
COL6A3
CSMD3
DSG1
ADGRG2
Percent of cases
Frame_Shift_Del
Frame_Shift_Ins
Missense_Mutation
Multi_Hit
Nonsense_Mutation
Splice_Site
Translation_Start_Site

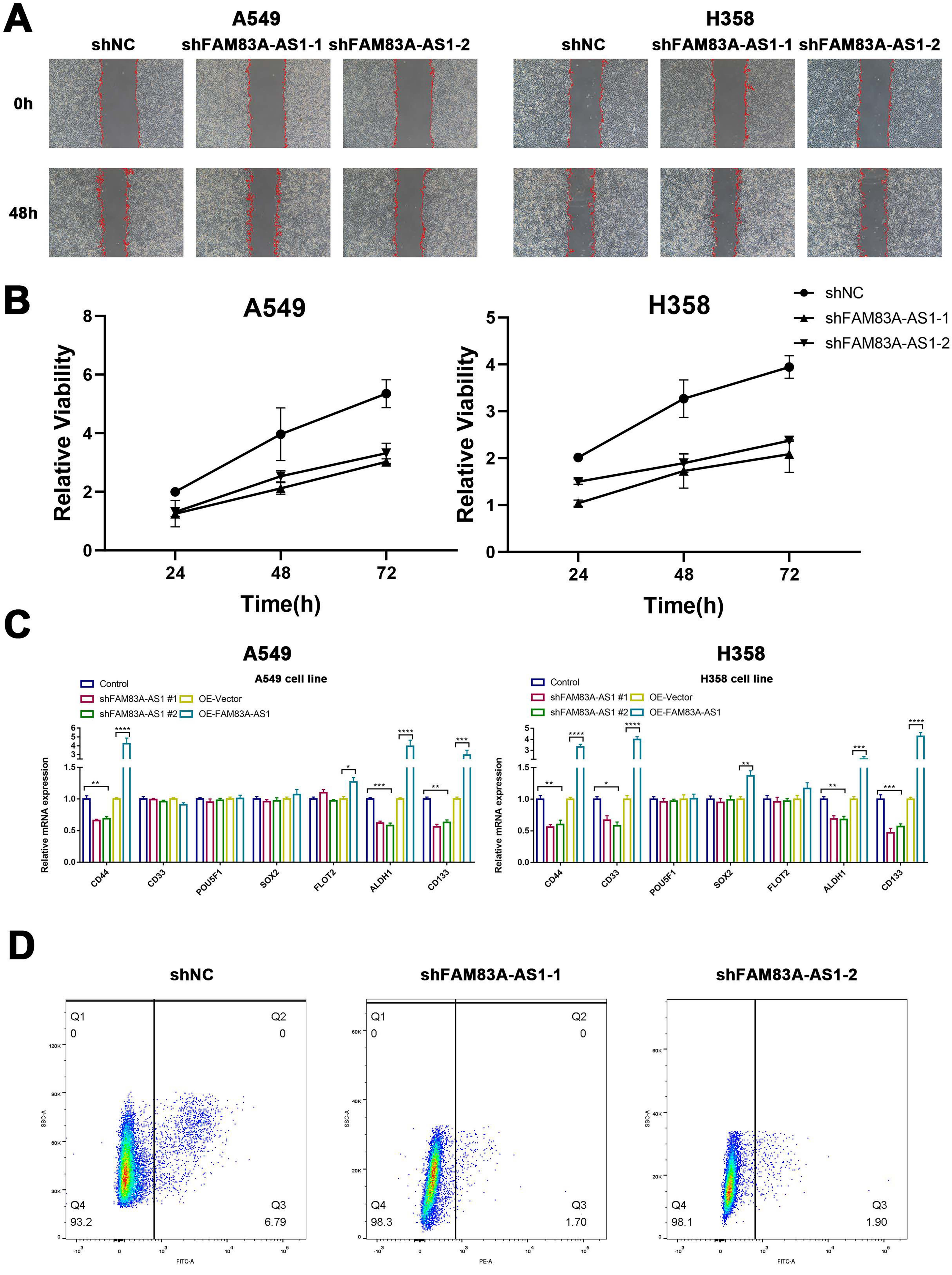
A
A549
H358
shNC
shFAM83A-AS1-1
shFAM83A-AS1-2
0h
48h
B
A549
H358
shNC
shFAM83A-AS1-1
shFAM83A-AS1-2
Relative Viability
Time(h)
24
48
72
C
A549
H358
A549 cell line
H358 cell line
Control
shFAM83A-AS1 #1
shFAM83A-AS1 #2
OE-Vector
OE-FAM83A-AS1
Relative mRNA expression
CD44
CD33
POU5F1
SOX2
FLOT2
ALDH1
CD133
D
shNC
shFAM83A-AS1-1
shFAM83A-AS1-2
Q1
0
Q2
0
Q4
93.2
Q3
6.79
Q4
98.3
Q3
1.70
Q4
98.1
Q3
1.90
SSC-A
FITC-A
PE-A

shNC

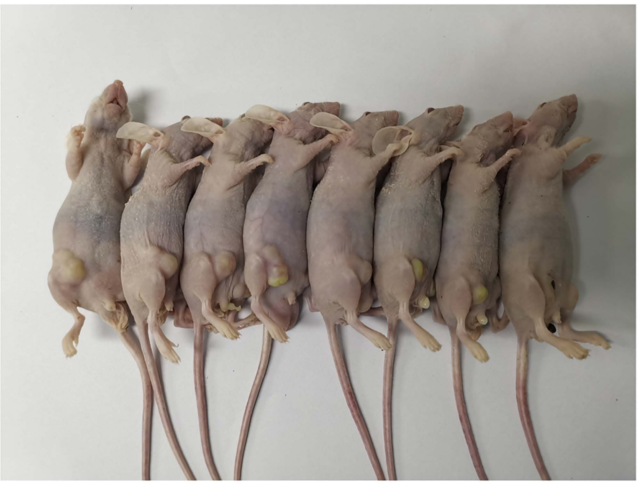

shFAM83A-AS1-1

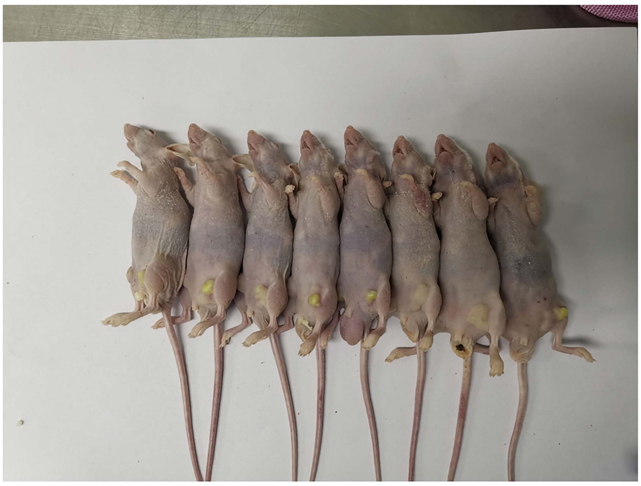

shFAM83A-AS1-2

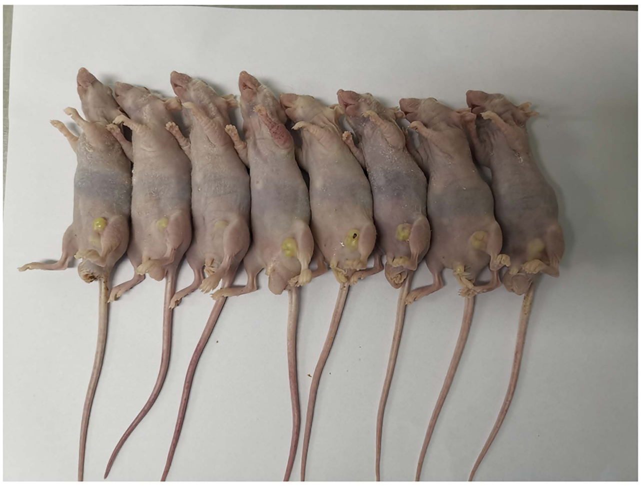

shFAM83A-AS1-2

shFAM83A-AS1-1

shNC

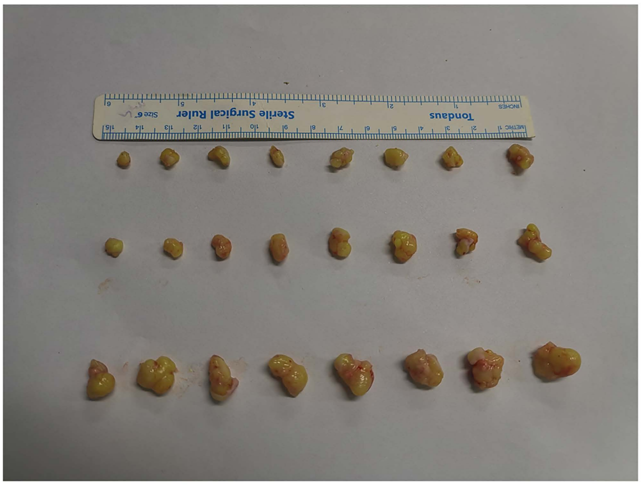

CD44

Forward Primer	CTGCCGCTTTGCAGGTGTA
Reverse Primer	CATTGTGGGCAAGGTGCTATT

CD33

Forward Primer	GGTGTGACTACGGAGAGAACC
Reverse Primer	GGTAGGGTGGGTGTCATTCC

POU5F1

Forward Primer	CAAAGCAGAAACCCTCGTGC
Reverse Primer	TCTCACTCGGTTCTCGATACTG

SOX2

Forward Primer	TGGACAGTTACGCGCACAT
Reverse Primer	CGAGTAGGACATGCTGTAGGT

FLOT2

Forward Primer	GCAGATTGCCGAGGGTGAAA
Reverse Primer	CTCCCCGATTTTGCGGATCTT

ALDH1

Forward Primer	CGGGAAAAGCAATCTGAAGAGGG
Reverse Primer	GATGCGGCTATACAACACTGGC

CD133

Forward Primer	AGTCGGAAACTGGCAGATAGC
Reverse Primer	GGTAGTGTTGTACTGGGCCAAT

SLC2A1

Forward Primer	TCTGGCATCAACGCTGTCTTC
Reverse Primer	CGATACCGGAGCCAATGGT

HK2

Forward Primer	TGCCACCAGACTAAACTAGACG
Reverse Primer	CCCGTGCCCACAATGAGAC

PFKL

Forward Primer	GTACCTGGCGCTGGTATCTG
Reverse Primer	CCTCTCACACATGAAGTTCTCC

GAPDH

Forward Primer	ACAACTTTGGTATCGTGGAAGG
Reverse Primer	GCCATCACGCCACAGTTTC

ENO1

Forward Primer	TGGTGTCTATCGAAGATCCCTT
Reverse Primer	CCTTGGCGATCCTCTTTGG

PDK1

Forward Primer	CTGTGATACGGATCAGAAACCG
Reverse Primer	TCCACCAAACAATAAAGAGTGCT

PKM2

Forward Primer	ATAACGCCTACATGGAAAAGTGT
Reverse Primer	TAAGCCCATCATCCACGTAGA

LDHA

Forward Primer	TTGACCTACGTGGCTTGGAAG
Reverse Primer	GGTAACGGAATCGGGCTGAAT

LDHB

Forward Primer	TCTGTGACCGCCAATTCTAAGA
Reverse Primer	GCACCAGATTGAGCCGACTC

MCT4

Forward Primer	CGGCTTTGTGCTTTACGCC
Reverse Primer	GCTGAAGAGGTAGACGGAGTA
